# LncRNA ZFAS1 contributes to osteosarcoma progression via miR-520b and miR-520e-mediated inhibition of RHOC signaling

**DOI:** 10.1016/j.clinsp.2022.100143

**Published:** 2022-12-05

**Authors:** Xiaofeng Liu, Mingyang Wang, Liwen Zhang, Lei Huang

**Affiliations:** Department of Orthopaedics, The First Affiliated Hospital of Dalian Medical University, Liaoning, People's Republic of China

**Keywords:** Osteosarcoma, LncRNA ZFAS1, miR-520b, miR-520e, RHOC, Apoptosis, Migration, Invasion

## Abstract

**Objectives:**

We examined the expression of Lnc-ZFAS1 in osteosarcoma and comprehensively evaluated its effects on osteosarcoma *in vitro* and *vivo*. Moreover, we revealed the regulatory mechanism between Lnc-ZFAS1 and miR-520b/miR-520e-mediated RHOC and provided a novel clue for ameliorating osteosarcoma.

**Method:**

The expression of Long non-coding RNA Zinc Finger Antisense 1 (LncRNA ZFAS1) osteosarcoma tissues and normal tissues in the TCGA database was analyzed. Then, LncRNA ZFAS1 expression was further verified in clinical samples and osteosarcoma cell lines (U2OS and KHOS), as well as the human osteoblast cell line hFOB1.19 by qRT-PCR. Thereafter, LncRNA ZFAS1 was overexpressed or silenced to explore its effects on cell proliferation, apoptosis, migration, invasion, and Epithelial-Mesenchymal Transition (EMT). The fundamental mechanism through which Lnc-ZFAS1 affects osteosarcoma progression was further investigated and verified.

**Results:**

We found that LncRNA ZFAS1 was upregulated in osteosarcoma, and Lnc-ZFAS1 overexpression facilitated osteosarcoma cells proliferation, migration, invasion and EMT, while Lnc-ZFAS1 silence exerted reverse influence. Mechanistically, Lnc-ZFAS1 functionally acted as a sponger of microRNA-520b (miR-520b) and microRNA-520e (miR-520e) to up-regulate Ras Homologue C (RHOC). In addition, depleted Lnc-ZFAS1 restrained osteosarcoma cells proliferation, migration, and invasion, which could be rescued by RHOC overexpression. Lnc-ZFAS1 was upregulated in osteosarcoma and Lnc-ZFAS1 could exert promoted impact upon osteosarcoma cells proliferation, migration, invasion, and EMT *in vitro*.

**Conclusions:**

Lnc-ZFAS1 acted sponger of miR-520b and miR-520e to promote RHOC, indicating that Lnc-ZFAS1/miR-520b/RHOC and Lnc-ZFAS1/miR-520e/RHOC axes might serve as potential therapeutic strategies against osteosarcoma.

## Highlights

• Lnc-ZFAS1 is highly expressed in osteosarcoma.
• Lnc-ZFAS1 could promote cell proliferation, migration, invasion and EMT *in vitro*.
• Lnc-ZFAS1 acted sponger of miR-520b and miR-520e to promote RHOC.

## Introduction

Osteosarcoma, also known as osteogenic sarcoma, is verified as the most prevalent type of malignant bone tumor, frequently occurring in adolescents or children under the age of 20.^1^^,^^2^ Osteosarcoma (OS) is the most malignant primary bone tumor originating from primary osteoblasts, which is characterized by the production of osteoid substances.^3^ Clinically, osteosarcoma is pathologically characterized by lower limb pain, lumps, anemia, and even toddle.^4^^,^^5^ Although the overall survival rate has been significantly ameliorated due to the early diagnosis and timely treatment, osteosarcoma still remains high mortality in children and adolescents.^6^ Thus, uncovering the prognostic genes predicting the occurrence and progression of osteosarcoma has far-reaching and comprehensive significance.

Long non-coding RNA (LncRNA) is a type of RNA molecule with over 200 nucleotides in length.^7^^,^^8^ Owning to the conserved secondary structure, lncRNA has been uncovered to interact with proteins, DNA and RNA,^9^ and functionally contribute to multiple biological processes, especially in cellular activities, like proliferation, apoptosis, migration, and invasion. More specifically, Wang X et al. stated that lnc-CTSLP8 could efficiently facilitate cell migration and invasion in ovarian cancer by functionally acting as an endogenous.^10^ Zhang C et al. indicated that LncRNA-ALC could epigenetically downregulate LZTS1 in a DNA methylation-dependent manner, thereby promoting cell metastasis in colorectal cancer.^11^ In contrast, Pan and his colleagues found that lnc-CTSLP4 exerted a repressive influence upon gastric cancer cells metastasis and EMT progress via inhibiting HNRNPAB-mediated Snail transcription.^12^ LncRNA ZFAS1 (Lnc-ZFAS1) has been verified to facilitate the progression of osteosarcoma according to Liu G^13^ and Zhao Z's research.^14^ However, the Lnc-ZFAS1-mediated mechanism in osteosarcoma still remains insufficient.

Recently, the dysregulation of miRNAs has been verified closely correlated with tumors growth and metastasis,^15^ and accumulating studies have proved the crucial role of miRNAs in osteosarcoma, like miR-411-5p,^2^ miR-539^16^ and miR-4660.^17^ Liu W et al. suggested miR-140 could weak the protein stabilization of LSD1 mediated by targeting USP22 and facilitate the expression of p21 to block osteosarcoma progression.^18^ Interestingly, Wang J et al. have reported the suppressive influence of miR-520b on growth and metastasis in osteosarcoma.^19^ However, the role of miR-520b/miR-520e in osteosarcoma and the potential regulatory mechanism between miR-520b/miR-520e and lncRNAs in osteosarcoma has not been revealed.

The Ras Homologue (RHO) family is a group of Guanosine Triphosphate (GTP) binding proteins with approximately 20‒25 KD relative molecular mass.^20^ RHO consists of 20 members and is generally named RHO GTPases due to the GTP activity and is classified into 5 subfamilies.^21^ It has been proved that the RHO family plays an indispensable role in the signal transduction^22^ and cellular activities,^23^ such as proliferation, apoptosis, transcription, transformation, infiltration and metastasis of malignant tumor cells. As a member of the RHO family, Ras Homologue C (RHOC) has been proven to participate in the occurrence and progression of osteosarcoma,^24^^,^^25^ whereas whether Lnc-ZFAS1 mediates RHOC in osteosarcoma remains unclear.

Thus, in this work, we examined the expression of Lnc-ZFAS1 in osteosarcoma and comprehensively evaluated its effects on osteosarcoma *in vitro*. Moreover, we revealed the regulatory mechanism between Lnc-ZFAS1 and miR-520b/miR-520e-mediated RHOC and provided a novel clue for ameliorating osteosarcoma.

## Materials and methods

### Tissue collection

Osteosarcoma and corresponding normal tissues (n = 8) were respectively collected from the First Affiliated Hospital of Dalian Medical University, and the research strategy was formulated in accordance with the Declaration of Helsinki and was authorized by the First Affiliated Hospital of Dalian Medical University ethics committee. Signed informed consent was obtained from the patients.

### Cell culture and transfection

Osteosarcoma cell lines U2OS and KHOS, and human osteoblast cell line hFOB1.19 were obtained from ATCC (American Type Culture Collection, USA). Based on ATCC's instructions, these cell lines were incubated for further investigation.

The pcDNA-ZFAS1, si-ZFAS1-1, si-ZFAS1-2, miR-520b mimics, miR-520e mimics and pcDNA-RHOC were provided by Gene Chem (Shanghai, China). U2OS and KHOS cells were conducted transfection according to lipofectamine's (11668-019, Invitrogen, USA) protocols.

### Colony formation assay

To evaluate the colony formation ability, a 6-well plate was utilized for the incubation of U2OS and KHOS cells. After 10 days of culture, the transfected cells were respectively conducted fixation and stained by paraformaldehyde and crystal violet. The colonies were counted using a microscope.

### EDU

For the EdU assay, U2OS and KHOS cells were mixed with EdU reagent in accordance with instructions of An Edu assay kit (KeyGen Biotech, Nanjing), followed by counterstaining using Hoechst 33342. A light microscope was utilized for the visualization and measurement.

### Transwell

For the migration and invasion estimation, the upper chamber was employed for a culture of U2OS and KHOS cells, while the lower chamber was replenished with 10% FBS. After incubation, cells were removed from the upper chamber and the migratory or invasive cells were counted.

### Wound healing

To establish scratched cells, U2OS and KHOS cells were maintained in 6-well plates for 24h, followed by forming the wound using pipette tips, and then these cells were incubated for 24h. The width of the scratch was detected. The images were photographed using a microscope.

### Luciferase reporter assay

To verify the interaction between miR-520b and miR-520e with Lnc-ZFAS1 and RHOC, a luciferase reporter assay was performed in KHOS cells. In brief, the Wild-Type (WT) and Mutant (Mut) binding sites of the 3′-untranslated regions of Lnc-ZFAS1 and RHOC for miR-520b and miR-520e were amplified using PCR. The WT and Mut 3′-UTRs were introduced into the pmirGLO luciferase expression vector (Promega Corporation) to construct the following plasmids, Lnc-ZFAS1-WT, Lnc-ZFAS1-MUT, RHOC-WT and RHOC-MUT. KHOS cells were co-transfected with 2.5 µg pmirGLO-Lnc-ZFAS1-WT, pmirGLO-Lnc-ZFAS1-MUT, pmirGLO-RHOC-WT or RHOC-MUT and 50 nM miR-520b mimics, miR-520e mimics, or NC-mimics using Lipofectamine 3000 at 37°C. At 48h post-transfection, luciferase activities were measured using the dual-luciferase reporter assay system (Promega Corporation) according to the manufacturer's protocol. Firefly luciferase activities were normalized to Renilla luciferase activities.

### RNA-pull down

Sense or antisense Lnc-ZFAS1 sequences were marked with biotin and then were cultured with protein lysis extracted from KHOS cells lysate, followed by incubating with agarose beads (Invitrogen) coupled with streptavidin. The q-PCR was employed to detect the amounts of Lnc-ZFAS1, miR-520b, miR-520e, and miR-934.

### Immunofluorescence staining

For the detection of F-actin, transfected KHOS cells were incubated in the coverslips of six-well plates overnight, followed by mixing with 100 µg/mL PCFE and 1000 µg/mL dacarbazine for 24h. After fixing with methanol, cells were cultured with primary anti-F-actin antibody (ab205, abcam, UK) for 1h, and then incubated with secondary antibodies. A fluorescence microscope (Leica DM4000 B, Germany) was utilized for the visualization.

### Subcellular fractionation localization

To verify the location of Lnc-ZFAS1, KHOS cells were performed the isolation of cytoplasmic and nuclear RNA in accordance with the protocol of PARIS Kit (Life Technologies, USA). qRT-PCR was employed to examine the total RNA extracted from the cytoplasm and nucleus.

### RNA FISH

fluorescein amidite-labeled Lnc-ZFAS1 probes were designed and synthesized by RiboBio. A FISH Kit (RiboBio) was used to detect probe signals according to the manufacturer's instructions. Nuclei were stained with DAPI. All images were acquired on a confocal microscope system.

### RNA immunoprecipitation (RIP)

By using a Magna RNA-binding protein immunoprecipitation kit (Millipore, USA), RIP was performed to evaluate the correlation between Lnc-ZFAS1 and miR-520e/miR-520b. KHOS cells were centrifuged and washed prior to mixing with RIP lysis buffer. The lysates were harvested to incubated with RNAs magnetic beads that conjugated Anti-AGO2 antibody (ab186733,1:50, Abcam, UK) or anti-IgG (ab172730, 1:100, Abcam, UK). After treating samples with Proteinase K for 30 min with gentle agitation, immunoprecipitated RNA was isolated using TRIzol. Finally, co-precipitated RNAs were identified and analyzed via qRT-PCR.

### Western blotting (WB)

Protein samples extracted by RIPA buffer were isolated using SDS-PAGE, and were transferred into PVDF membranes, followed by incubating with primary antibodies. Anti-rabbit antibody coupled with peroxidase was determined as the secondary antibody. ECL reagent (Millipore) was utilized for the visualization of antigen-antibody reaction. The primary antibodies caspase-3 (ab32351, 1:5000), cleaved caspase-3 (ab32042, 1:500), caspase-9 (ab32539, 1:1000), cleaved caspase-9 (ab2324, 1 µg/mL), bax (ab32503,1:2000), Bcl-2 (ab32124, 1:1000), Cyclin D1 (ab16663, 1:50), E-cadherin (ab76055, 1:200), N-cadherin (ab76011, 1:5000), Vimentin (ab92547, 1:2000), MMP-9 (ab76003, 1:10000) were purchased from Abcam (UK). GAPDH (ab8245, 1:1000, abcam, UK) was considered as the normalization.

### qRT-PCR

Total RNA samples extracted by TRIzol reagent were transcribed to cDNA following the protocols of PrimeScript RT Reagent Kit (Takara, Dalian, China). Then SYBR Premix Ex Taq (Takara) system was employed for the analysis of real-time PCR. The relative expression was performed normalization with GAPDH and U6. The corresponding primer sequences are listed in Supplementary Table 1.

### Statistical analysis

In this research, SPSS 20.0 (Chicago, USA) and GraphPad Prism 7 (GraphPad Software, USA) were utilized for all data-related collation. The data were represented as the mean ± SD. The differences between internal groups were analyzed by Student's *t*-test and ANOVA; p < 0.05 indicates statistical significance.

## Results

### Lnc-ZFAS1 is highly expressed in osteosarcoma

**Figure 1:**
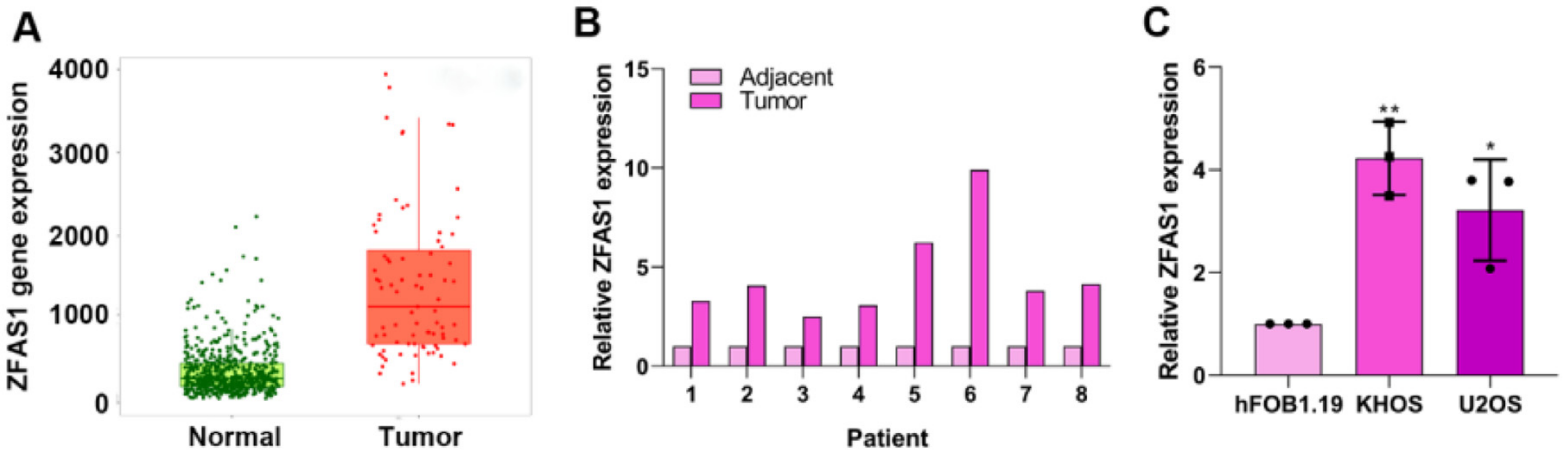
Lnc-ZFAS1 is upregulated in osteosarcoma. (A) TCGA database was used to evaluate the expression of Lnc-ZFAS1 in osteosarcoma tissues and normal tissues. (B) qRT-PCR was employed to detect the expression of Lnc-ZFAS1 in osteosarcoma tissues and normal tissues (n = 8). (C) qRT-PCR was utilized to measure the Lnc-ZFAS1 expression in osteosarcoma cell lines (U2OS and KHOS), and the human osteoblast cell line hFOB1.19 (*p < 0.05, **p < 0.01).

To explore the role of Lnc-ZFAS1 in osteosarcoma, we first analyzed the expression level of Lnc-ZFAS1 in osteosarcoma tissues and normal tissues in TCGA database. Lnc-ZFAS1 was upregulated in osteosarcoma tissues (Fig. 1A), which was further confirmed in clinic samples by q-PCR analysis (n = 8, Fig. 1B). In addition, we detected the expression level of Lnc-ZFAS1 in osteosarcoma cell lines U2OS and KHOS, as well as the human osteoblast cell line hFOB1.19 and found the expression of Lnc-ZFAS1 was significantly higher in U2OS and KHOS cells than in hFOB1.19 cell (Fig. 1C). Collectively, these results suggest that Lnc-ZFAS1 is highly expressed in osteosarcoma.

### Lnc-ZFAS1 facilitates cells proliferation and represses apoptosis in osteosarcoma

**Figure 2:**
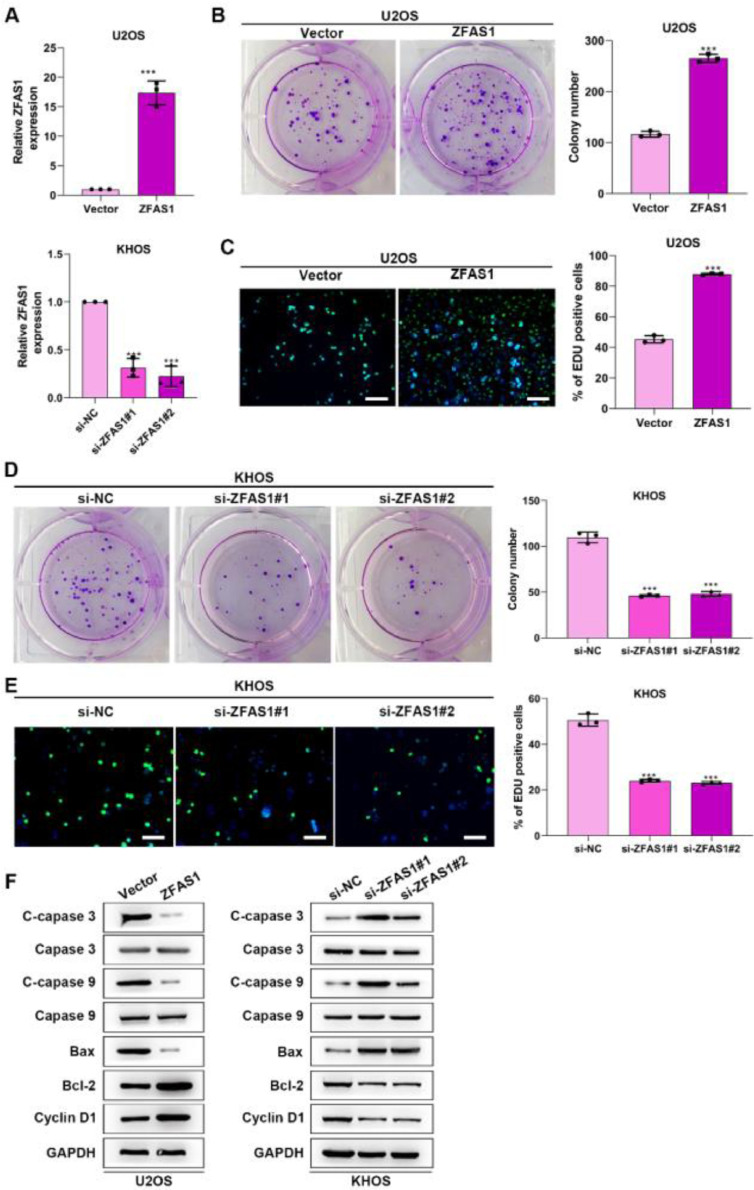
Lnc-ZFAS1 facilitates cell proliferation and represses apoptosis in osteosarcoma. For functional investigation, U2OS and KHOS cells were respectively infected with Lnc-ZFAS1 overexpression and Lnc-ZFAS1 shRNA plasmids, and qRT-PCR was performed for examination of transfected efficacy (A). Colony formation (B and D) and EdU (C and E) assays were utilized for proliferation analysis. (F) Western blotting was adopted to examine the expression of apoptosis-related proteins (**p < 0.01).

To uncover the functional influence of Lnc-ZFAS1 upon osteosarcoma progression, we established Lnc-ZFAS1-upregulated U2OS cells and Lnc-ZFAS1-downregulated KHOS cells (Fig. 2A). Lnc-ZFAS1 overexpression resulted in an obvious rise in (Fig. 2B) the cell proliferation of U2OS cells, as confirmed by colony formation assay (Fig. 2B) and EdU assay (Fig. 2C), while Lnc-ZFAS1 depletion caused reverse effect on KHOS cells proliferation (Fig. 2D‒E). In addition, upregulated Lnc-ZFAS1 enhanced the expression of anti-apoptosis proteins Bcl-2 and Cyclin D1 in U2OS cells, and exerted repressive effect upon pro-apoptosis proteins, including Bax, cleaved caspase-3 and cleaved caspase-9 (Fig. 2F). In contrast, depleted Lnc-ZFAS1 efficiently suppressed anti-apoptosis proteins and promoted pro-apoptosis proteins in KHOS cells. Overall, these findings suggested that Lnc-ZFAS1 facilitates osteosarcoma cell proliferation and restrains its apoptosis.

### Lnc-ZFAS1 induces osteosarcoma cells migration, invasion and EMT

**Figure 3:**
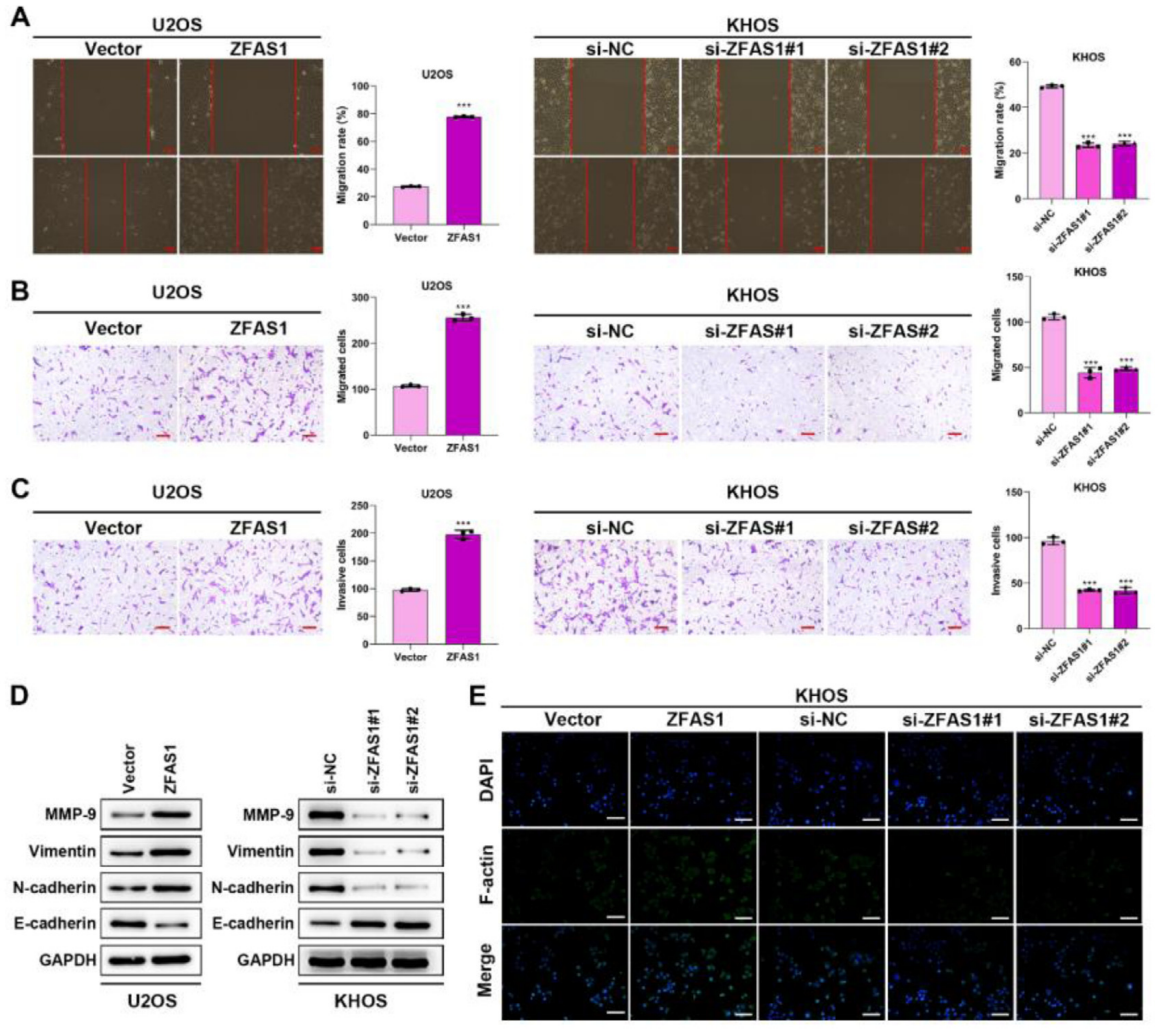
Lnc-ZFAS1 facilitates migration, invasion and EMT in osteosarcoma cells. Wound healing (A) and transwell (B and C) assays were adopted to examine the migratory and invasive capacities. (D) Western blotting was performed for the expression of EMT-related proteins. (E) F-actin was detected using Immunofluorescence staining (**p < 0.01).

We further investigated the effects of Lnc-ZFAS1 on osteosarcoma cell migration and invasion. Wound healing (Fig. 3A) and transwell (Fig. 3B‒C) assays demonstrated that Lnc-ZFAS1 depletion could shorten the wound gap and restrain the migratory and invasive capacities in KHOS cells, whereas Lnc-ZFAS1 overexpression induced promoted influence. Moreover, the up-regulation of MMP-9, Vimentin and N-cadherin and the downregulation of E-cadherin were observed in Lnc-ZFAS1 overexpressed U2OS cells, and the contrast results were induced by Lnc-ZFAS1 depletion, implying Lnc-ZFAS1’ promotion effect on EMT in osteosarcoma (Fig. 3D). In addition, Immunofluorescence staining assay exhibited the strong fluorescence intensity of F-actin in Lnc-ZFAS1-upregulated U2OS cells and the weak fluorescence intensity in Lnc-ZFAS1-downregulated KHOS cells (Fig. 3E). Taken together, our findings further confirmed the accelerated role of Lnc-ZFAS1 in osteosarcoma cells migration, invasion, and EMT.

### Lnc-ZFAS1 acts as a sponger of miR-520b and miR-520e in osteosarcoma

**Figure 4:**
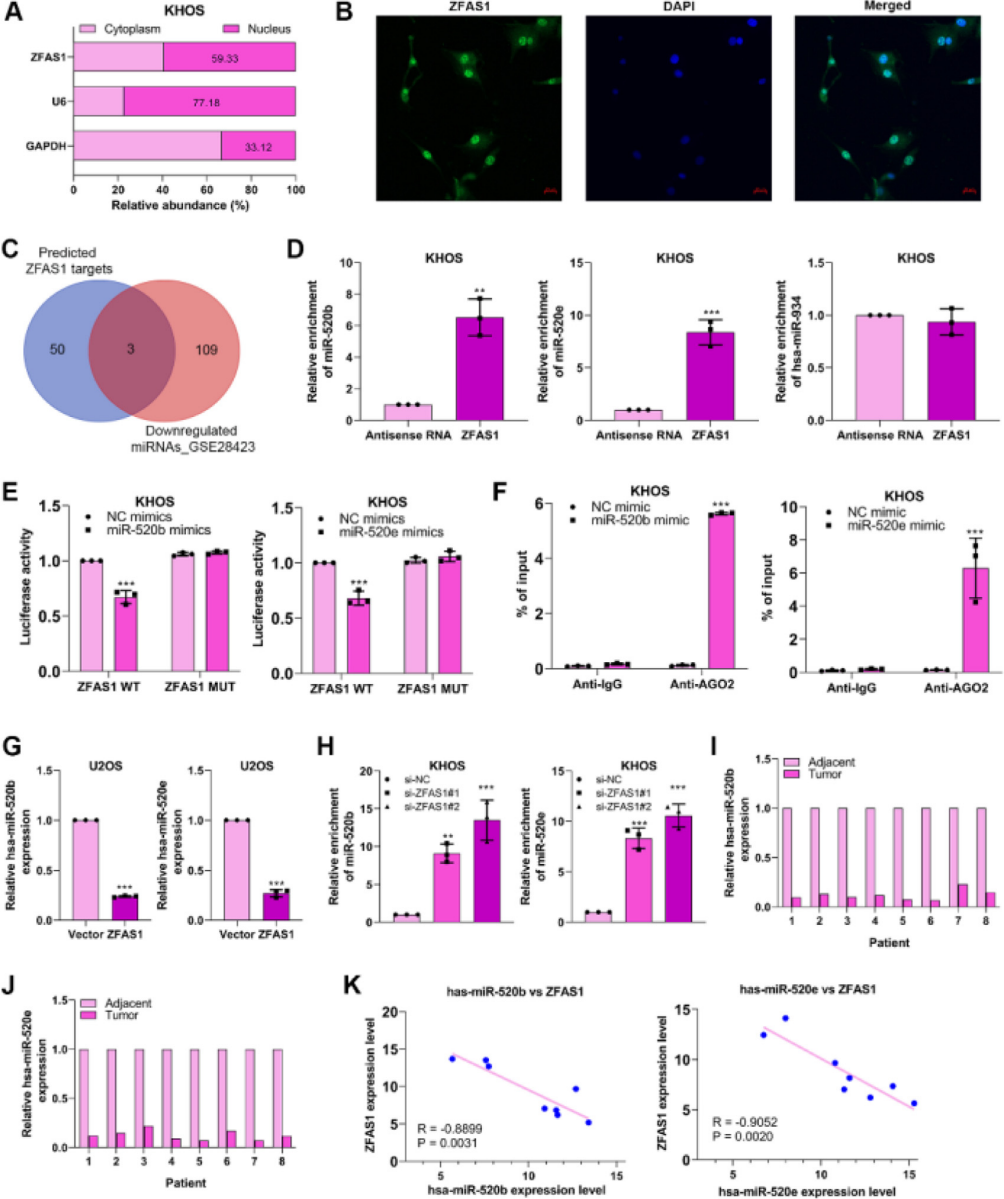
Lnc-ZFAS1 sponges miR-520b and miR-520e in osteosarcoma. (A) The subcellular location of Lnc-ZFAS1 was determined. GAPDH and U6 were respectively determined as the markers of cytoplasm and nucleus. (B) FISH results show Lnc-ZFAS1 (in green) cellular distribution in KHOS cells. (C) Starbase and GSE28423 databases were performed to obtain intersected hsa-miR-520b, hsa-miR-520e and hsa-miR-934. RNA pull-down (D), Luciferase reporter (E) and RIP (F) assays were utilized to explore the binding between Lnc-ZFAS1 with two miRNAs. (G) miR-520b and miR-520e were detected in Lnc-ZFAS1-upregulated U2OS cells and Lnc-ZFAS1-downregulated KHOS cells by qRT-PCR. (H) qRT-PCR was employed to detect the expression of miR-520b and miR-520e in osteosarcoma tissues and normal tissues (n = 8). (I) The correlation analysis between Lnc-ZFAS1 with two miRNAs (*p < 0.05, **p < 0.01).

To expose the fundamental mechanism through which Lnc-ZFAS1 affects osteosarcoma progression, we first examined the subcellular location of Lnc-ZFAS1 in KHOS cells and found Lnc-ZFAS1 was evenly enriched in the and nucleus (Fig. 4A). Notably, this was corroborated by the result of the FISH assay (Fig. 4B) Subsequently, we referred to Starbase and GSE28423 databases to explore the intersected miRNAs, which could potentially interacted with Lnc-ZFAS1. Among miRNAs downregulated in osteosarcoma, hsa-miR-520b, hsa-miR-520e and hsa-miR-934 was identified as the potential target of Lnc-ZFAS1 (Fig. 4C).

RNA pull-down results showed that biotin-labeled Lnc-ZFAS1 efficiently enriched miR-520b and miR-520e from KHOS cells lysate (Fig. 4D). Additionally, miR-520b mimics and miR-520e mimics respectively suppressed the luciferase activity of Lnc-ZFAS1 wild-type in KHOS cells, but not mutant Lnc-ZFAS1 3′-UTR (Fig. 4E). RIP assay further confirmed that anti-AGO2 complexes significantly enriched Lnc-ZFAS1 in miR-520b-upregulated and miR-520e-upregulated KHOS cells, respectively (Fig. 4F). To further validate the regulatory correlation between Lnc-ZFAS1 and miR-520b/miR-520e, we detected the expression of miR-520b and miR-520e in Lnc-ZFAS1-upregulated and downregulated cells and found that Lnc-ZFAS1 overexpression suppressed these two miRNAs expression, while Lnc-ZFAS1 depletion elevated them (Fig. 4G). Furthermore, we observed that both miR-520b and miR-520e were lowly expressed in osteosarcoma tissues compared with that in normal tissues (n = 8, Fig. 4H), and their expression levels were negatively associated with Lnc-ZFAS1 expression (Fig. 4I). Collectively, our data demonstrated that Lnc-ZFAS1 might function as a ceRNA to sponge miR-520b and miR-520e in osteosarcoma.

### miR-520b and miR-520e negatively modulates RHOC in osteosarcoma

**Figure 5:**
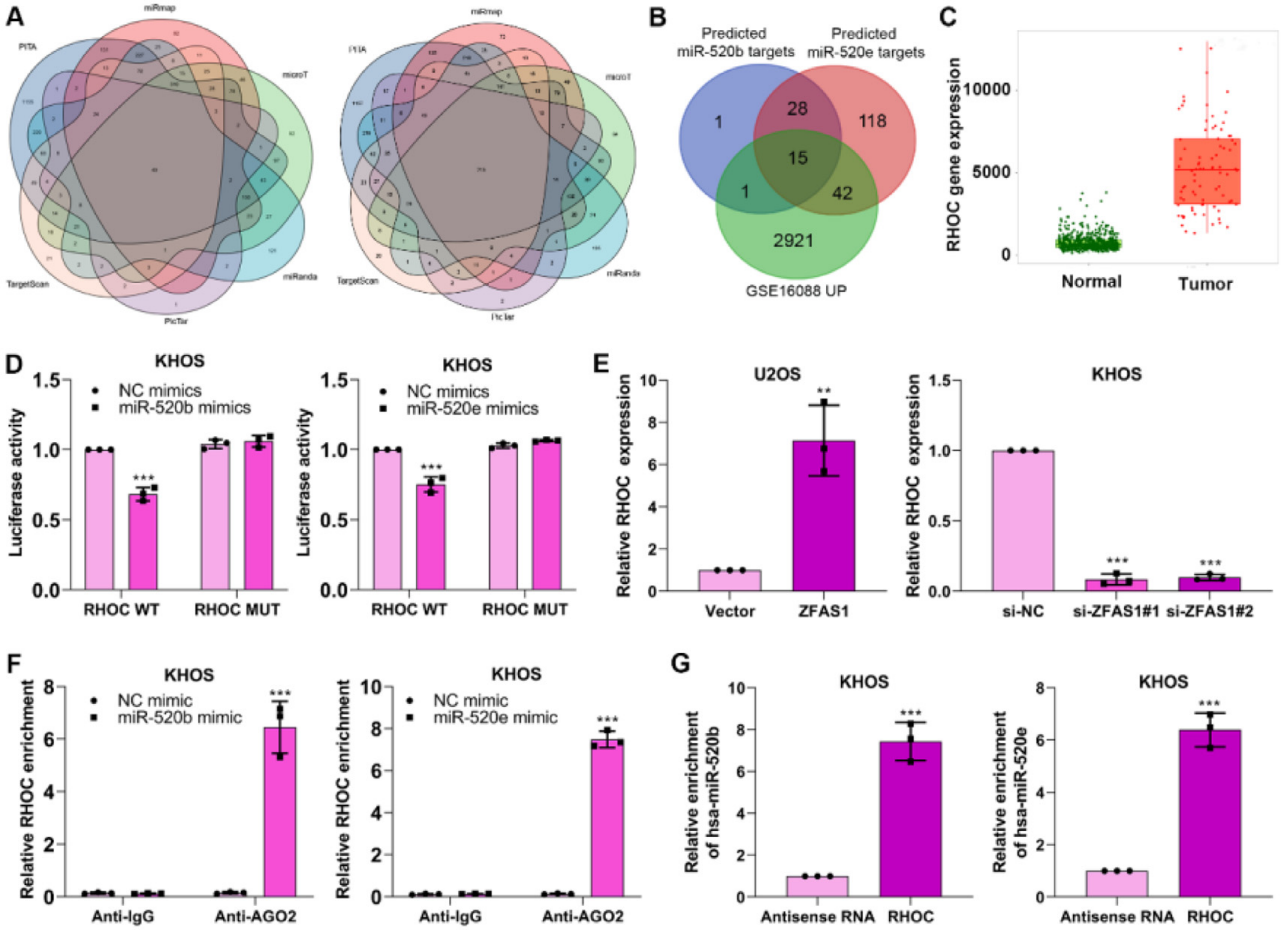
miR-520b and miR-520e negatively modulates RHOC in osteosarcoma. (A) PITA, miRmap, microT, miRanda, PicTar and TargetScan databases were performed to predict the target genes of miR-520b and miR-520e. (B) The intersection genes were obtained through intersecting predicted genes and GSE16088 database. (C) Target database was utilized to analyze the different expression of RHOC in osteosarcoma. Dual luciferase reporter (D), q-PCR (E), RIP (F) and RNA pull-down (G) assays were performed to validate the correlation between miR-520b or miR-520e and RHOC.

We aimed to reveal the specific mechanism of miR-520b and miR-520e in osteosarcoma progression. Bioinformatics databases including PITA, miRmap, microT, miRanda, PicTar and TargetScan were performed to predict the target genes of miR-520b and miR-520e (Fig. 5A). The predicted target genes of miR-520b and miR-520e and the differentially up-regulated genes in GSE16088 database were intersected15 intersection genes, including RHOC, BAMBI, UBE2J1, SPOP, TARDBP, SUCO, TLE4, MEF2C, TIPARP, UBE2B, ATAD2, NFIB, DPP8, ARID4B, APP (Fig. 5B) were finally identified. Among them, RHOC has been proven to involve in the progression of osteosarcoma,^25^ thus we determined RHOC as the target gene in further research.

To investigate the correlation between RHOC and two miRNAs, we first analyzed the expression of RHOC in osteosarcoma, based on the Target database, we observed RHOC was upregulated in osteosarcoma tissues (Fig. 5C). Next, dual luciferase reporter assay exhibited that miR-520b mimics and miR-520e mimics considerably restrained the luciferase activity in RHOC-WT plasmid transfected KHOS cells, compared to that in RHOC-MUT plasmid-transfected cells (Fig. 5D). Furthermore, Lnc-ZFAS1 overexpression efficiently facilitated the expression of RHOC in U2OS cells, while depleted Lnc-ZFAS1 exerted inhibited effect upon KHOS cells (Fig. 5E). Interestingly, we found that anti-AGO2 complexes could respectively enrich RHOC in miR-520b-upregulated and miR-520e-upregulated KHOS cells (Fig. 5F), and biotin-labeled RHOC could pull down miR-520b and miR-520e in an efficiently way (Fig. 5G). Together, these results indicate that miR-520b and miR-520e could negatively regulate RHOC in osteosarcoma.

### Lnc-ZFAS1 facilitates osteosarcoma progression via RHOC

**Figure 6:**
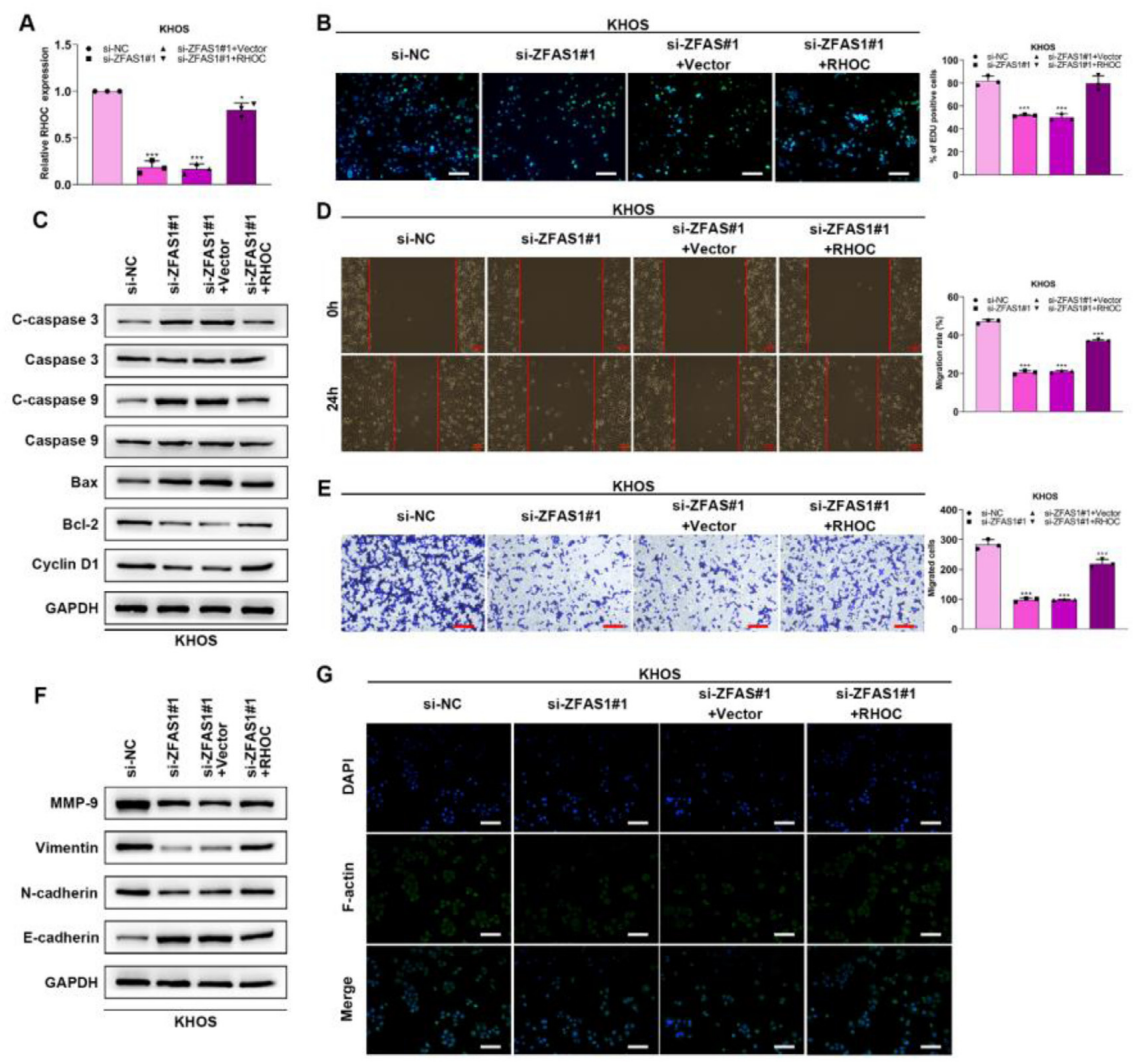
Lnc-ZFAS1 facilitates osteosarcoma progression via RHOC. To verify whether Lnc-ZFAS1 acts oncogenic driver in osteosarcoma progression in a RHOC-dependent manner, KHOS cells were transfected with Lnc-ZFAS1 siRNA and RHOC overexpression plasmids, qRT-PCR (A) was performed to detect the transfection efficacy. (B) EdU was performed for proliferation analysis. (C) Apoptosis-related proteins were detected by western blotting. Migration and invasion were evaluated by wound healing (D) and transwell (E). EMT-related proteins were measured by western blotting (F). (G) F-actin was examined using Immunofluorescence staining (*p < 0.05).

To verify whether Lnc-ZFAS1 acts oncogenic driver in osteosarcoma progression in an RHOC-dependent manner, we co-transfected Lnc-ZFAS1 siRNA and RHOC overexpression plasmids into KHOS cells (Fig. 6A). EdU assay exhibited that Lnc-ZFAS1 depletion strikingly induced the drop of EdU positive cells, which could be rescued by RHOC overexpression (Fig. 6B). In addition, upregulated RHOC restored the expression levels of anti-apoptosis proteins bcl-2 and Cyclin D1, which were inhibited by Lnc-ZFAS1 (Fig. 6C). Moreover, upregulating RHOC partly abrogated the impairment of the migratory and invasive capacities of KHOS cells caused by Lnc-ZFAS1 silence (Fig. 6D‒E). RHOC also blocked the EMT induced by Lnc-ZFAS1, which could be specifically embodied in the restoration of MMP-9, Vimentin and N-cadherin (Fig. 6F). Additionally, the inhibited effect on the fluorescence intensity of F-actin also could be rescued by RHOC overexpression (Fig. 6G). Our findings indicate that Lnc-ZFAS1 acted a functional role in osteosarcoma progression through targeting RHOC.

## Discussion

In this study, our findings suggested Lnc-ZFAS1 as an oncogenic driver in osteosarcoma progression. Moreover, we identified Lnc-ZFAS1 functionally acted as a sponger of miR-520b and miR-520e to upregulate RHOC in osteosarcoma. We demonstrated the Lnc-ZFAS1/miR-520b/miR-520e/RHOC axis involved in the development of osteosarcoma.

As an emerging member of Long non-coding RNAs, Lnc-ZFAS1 has been proven strongly correlated with enormous diseases, like myocardial infarction,^26^ lung injury^27^ and cardiovascular disease.^28^ In addition, accumulating research have exhibited the oncogenic role of Lnc-ZFAS1 in multiple cancers, including colorectal cancer,^29^ nasopharyngeal carcinoma^30^ and cervical cancer.^31^ Zhu T et al. suggested that Long non-coding RNA ZFAS1 facilitated the progression of gastric cardia adenocarcinoma through upregulating EPAS1.^32^ Consistently, our results demonstrated that Lnc-ZFAS1 was upregulated in osteosarcoma, and Lnc-ZFAS1 could significantly facilitate the osteosarcoma cells' proliferation, migration and invasion *in vitro*. In principle, EMT was identified as the transformation of epithelial to mesenchymal cells, imparting the capacities of migration and invasion of cells.^33^ Interestingly, we found Lnc-ZFAS1 induced the EMT in osteosarcoma. Similarly, O'Brien SJ and his colleagues revealed that long non-coding RNA ZFAS1 promoted the epithelial-mesenchymal transition in colon adenocarcinoma via sponging miR-200/ZEB1.^34^

It has been believed that LncRNAs interferences the expression of miRNAs by acting as a ceRNA,^35^ and the interaction between Lnc-ZFAS1 and miRNAs has been proved by extensive reports. For example, Liu J et al. demonstrated that LncRNA ZFAS1 sponged miR-3924 to mediate RHOA/ROCK2 pathway, thereby accelerating the growth and metastasis of pancreatic adenocarcinoma, and Zhu HL et al. revealed the regulatory mechanism between LncRNA ZFAS1 and miR-129, and suggested that LncRNA ZFAS1 could induce ovarian granulosa cells proliferation and endocrine disturbance in polycystic ovary syndrome via upregulating HMGB1.^36^ In addition, based on various mechanistic assays, such as luciferase reporter, RNA pull-down, RNA immunoprecipitation, and enormous experiments *in vitro* and *in vivo*, Lnc-ZFAS1 has been proved to act as a sponger of microRNA-135a,^14^ miR-892b^37^ and miR-193a-3p.^38^ In this work, we identified miR-520b and miR-520e as the target miRNAs of Lnc-ZFAS1 and found these two miRNAs were downregulated in osteosarcoma. Furthermore, we validated that Lnc-ZFAS1 could sponge miR-520b and miR-520e in osteosarcoma cell lines.

As referred to above, RHOC belongs to one of the subfamilies of RHO family, and is closely associated with diverse cancers, like breast cancer,^39^ human Glioma^40^ and prostate cancer.^41^ Here in, we determined RHOC as the target protein of miR-520b and miR-520e, and confirmed the negative correlation between RHOC with these two miRNAs based on multiple mechanistic assays in osteosarcoma cell lines. Moreover, we observed RHOC could act a dispensable role in recovering the impairment of the capacities of proliferation, migration, and invasion, as well as the loss of EMT caused by Lnc-ZFAS1, thereby identifying RHOC as an oncogenic driver in osteosarcoma.

Nevertheless, there are several limitations to this study. One limitation is that our results only demonstrated the roles of Lnc-ZFAS1 based on *in vitro* experiments, and further *in vivo* studies are required to perform in the future study. Besides, only eight patients, (a relatively small total sample size) were included in this study. Hence, our study should be viewed as hypothesis-generating, to be followed by larger prospective studies to confirm our findings.

To summarize, our paper concluded that Lnc-ZFAS1 was upregulated in osteosarcoma and Lnc-ZFAS1 could exert promoted impact upon osteosarcoma cells proliferation, migration, invasion and EMT *in vitro*. Moreover, Lnc-ZFAS1 acted sponger of miR-520b and miR-520e to promote RHOC. Our findings provided Lnc-ZFAS1/miR-520b/RHOC and Lnc-ZFAS1/miR-520e/RHOC axes as potential therapeutic strategies against osteosarcoma.

## Authors’ contributions

Xiaofeng Liu and Lei Huang designed the research plan. Xiaofeng Liu, Mingyang Wang, Liwen Zhang and Lei Huang performed the experiments. Lei Huang wrote the manuscript.

## Funding

No funding was received.

## Conflicts of interest

The authors declare no conflicts of interest.

## Appendix Supplementary materials

Image, application 1
