# Supplementary material for: LncRNA ZFAS1 contributes to osteosarcoma progression via miR-520b and miR-520e-mediated inhibition of RHOC signaling
Source: Clinics (Sao Paulo). 2022 Dec 5;78:100143. doi: 10.1016/j.clinsp.2022.100143 (PMC9727593; doi:10.1016/j.clinsp.2022.100143)
Supplement: Supplementary file 1 [file mmc1.docx]

CLINICS-D-22-00217 – Supplementary Material

**Supplementary Table 1** Sequences of PCR primers used in this study.

| **GAPDH** | Forward (5’-3’) | GGAGCGAGATCCCTCCAAAAT |
| --- | --- | --- |
|  | Reverse (5’-3’) | GGCTGTTGTCATACTTCTCATGG |
| **RHOC** | Forward (5’-3’) | GGAGGTCTACGTCCCTACTGT |
|  | Reverse (5’-3’) | CGCAGTCGATCATAGTCTTCC |
| **ZFAS1** | Forward (5’-3’) | CCGGAGTGTGGTACTTCTCC |
|  | Reverse (5’-3’) | CCAGAGGTCTCCAACGAAGA |
| **MiR-520b** | Forward (5’-3’) | AAAGTGCTTCCTTTTAGAGGG |
|  | Reverse (5’-3’) | GCGAGCACAGAATTAATACGAC |
| **MiR-520e** | Forward (5’-3’) | GAAAGTGCTTCCTTTTTAGGG |
|  | Reverse (5’-3’) | GTCCAGTTTTTTTTTTTTTTTCCTAC |

**Supplementary Table 2** The role of molecules studied in this study.

| **Molecules** | **Type of RNA** | **Role** |
| --- | --- | --- |
| Lnc-ZFAS1 | LncRNA | Oncogenic RNA; contributes to the proliferation, migration, invasion, and EMT process of osteosarcoma cells |
| MiR-520b | microRNA | Tumor suppressor; inhibits the proliferation, migration, invasion, and EMT process of osteosarcoma cells |
| MiR-520e |  |  |
| RHOC | mRNA | Oncogene; contributes to the proliferation, migration, invasion, and EMT process of osteosarcoma cells |
